# New Waterborne Polyurethane-Urea Synthesized with Ether-Carbonate Copolymer and Amino-Alcohol Chain Extenders with Tailored Pressure-Sensitive Adhesion Properties

**DOI:** 10.3390/ma13030627

**Published:** 2020-01-31

**Authors:** Mónica Fuensanta, Abbas Khoshnood, Francisco Rodríguez-Llansola, José Miguel Martín-Martínez

**Affiliations:** 1 Adhesion and Adhesive Laboratory, University of Alicante, 03080 Alicante, Spain; monica.fuensanta@ua.es (M.F.); abbas.khoshnood@ua.es (A.K.); 2 UBE Corporation Europe, Polígono El Serrallo, 12100 Grao Castellón, Spain; f.rodriguez@ube.com

**Keywords:** waterborne polyurethane-urea dispersion, pressure-sensitive adhesives, copolymer of ether and carbonate polyol, amino-alcohol chain extender, adhesion, viscoelastic properties, tack

## Abstract

New waterborne polyurethane-urea dispersions with adequate adhesion and cohesion properties have been synthesized by reacting isophorone diisocyanate, copolymer of ether and carbonate diol polyol and three amino-alcohols with different number of OH groups chain extenders using the prepolymer method. The waterborne polyurethane-urea dispersions were characterized by pH, particle-size distribution, and viscosity, and the polyurethane-urea films were characterized by attenuated total reflectance infrared (ATR-IR) spectroscopy, differential scanning calorimetry (DSC), thermal gravimetric analysis (TGA), and plate-plate rheology (temperature and frequency sweeps). Polyurethane-urea pressure-sensitive adhesives (PUU PSAs) were prepared by placing the waterborne polyurethane dispersions on polyethylene terephthalate (PET) films and they were characterized at 25 °C by creep test, tack and 180° peel test. The waterborne polyurethane-urea dispersions showed mean particle sizes between 51 and 78 nm and viscosities in the range of 58–133 mPa·s. The polyurethane-urea films showed glass transition temperatures (T_gs_) lower than −64 °C, and they showed a cross of the storage and loss moduli between −8 and 68 °C depending on the number of OH groups in the amino-alcohol chain extender. Different types of PUU PSAs (removable, high shear) were obtained by changing the number of OH groups in the amino-alcohol chain extender. The tack at 25 °C of the PUU PSAs varied between 488 and 1807 kPa and the 180° peel strength values ranged between 0.4 and 6.4 N/cm, and their holding times were between 2 min and 5 days. The new PUU PSAs made with amino-alcohol chain extender seemed very promising for designing environmentally friendly waterborne PSAs with high tack and improved cohesion and adhesion property.

## 1. Introduction

Polyurethane-urea (PUU) materials are versatile polymers commonly used as adhesives and coatings [1]. During the last few years, the development of eco-friendly polyurethane adhesives has become an important environmental and health concern. As compared to the conventional solvent-born polyurethane adhesives, waterborne polyurethane adhesives show lower levels of organic compounds and a lack of toxicity [2].

Waterborne polyurethane-urea materials are obtained by reacting polyol, diisocyanate, internal emulsifier and chain extender, and they show segmented structures consisting of soft (SS) and hard (HS) segments, as well as ionic interactions [3,4,5]. The hard and soft segments are thermodynamically incompatible, resulting in phase separation due to the formation of hard and soft microdomains in the PUUs, and the degree of phase separation determines their properties [6]. The soft segments are made of polyol and provide flexibility to the PUU, whereas the hard segments are made by reaction of the diisocyanate and the chain extender, and they impart mechanical properties. The ionic interactions of the pendant groups of the internal emulsifier in different polymeric chains also contribute to the mechanical properties of the PUU. Furthermore, both hard and soft segments can be organized in either amorphous or crystalline ordered domains by formation of hydrogen bonds between them, and this also determines the PUU properties [6].

Pressure-sensitive adhesives (PSAs) are polymeric materials which produce immediate physical bond to a substrate by the application of light pressure for a short time. PSAs are used in general in labels and tapes, post-its, packaging tapes, diapers, masking tapes, and medical bandages, etc. The requirements of the PSAs for these applications are good tack, adequate peel resistance, and high-shear resistance [7]. During bonding of a substrate by a PSA, good wetting and adequate adhesion are needed, but the PSA also needs to resist de-bonding forces by high cohesion and energy dissipation without leaving residues on the substrate, i.e., high cohesion is necessary. In other words, the PSAs require a balance between their viscoelastic properties, i.e., at low strain rates they must flow for imparting adequate bonding and tack, and at high strain rates they must have a high cohesion and elasticity for de-bonding. It should be noted that the adhesion and cohesion properties are opposite properties and they must be adequate in PSAs.

The viscoelastic properties of the PSAs can be tailored by using copolymers with segmented structure in which the hard domains impart the elastic properties and the soft domains control the viscous properties. In this sense, the PUUs have a potential for manufacturing PSAs but they are rarely used because of their low tack and low peel adhesive strength, i.e., insufficient adhesion and cohesion properties. However, the PUUs show good temperature and solvent resistance, and have good low temperature performance—properties that are desirable in PSAs [8].

Most PSAs are obtained from solvent-born formulations which, although effective, have environmental and health concerns, limiting their application in food and medical areas. Therefore, there is a need for developing waterborne-based PSAs. There are some previous studies on the development of waterborne polyurethane dispersions intended for PSAs, and the most of them are based on the use of hydroxyl-terminated polybutadiene (HTPB) polyols. Czech et al. [9] synthesized water-dispersible polyurethane PSAs containing hydroxylated polybutadienes, and demonstrated that the addition of polypropylene glycol (PPG) of high molecular weight and dimethylolpropionic acid (DMPA), and an isocyanate cross-linking agent improved the mechanical properties of the PSAs. Lagiewczyk and Czech [10] developed aqueous polyurethane PSAs containing hydroxylated polybutadiene (HTPB) as self-adhesives for protective films and they found that the increase of both HTPB and PPG amounts increased the viscosity and the thermal and the mechanical properties of the PSAs, which showed low tack, low adhesion and excellent cohesion after cross-linking with selected multifunctional isocyanates. In another study, Czech et al. [11] synthesized waterborne polyurethane PSAs with HTPB, hydroxylated benzophenone, diisocyanates, high molecular weight PPG and dicarboxylic acids. Without crosslinker, the polyurethane PSAs exhibited low tack and peel adhesion, but the addition of small amounts of polycarbodiimide increased both tack and peel adhesion. On the other hand, waterborne polyurethane PSAs without crosslinker have been synthesized with PPGs of different molecular weights and hydroxyl-terminated polybutadienes [12], and an increase of the storage modulus was found when the molecular weight of the PPG increased, and an increase in tack resulted when the amount of polybutadiene increased. In recent research, Akram et al. [13,14] have studied the influence of the isocyanate and the polyol on the adhesion properties of waterborne polyurethane PSAs made with HTPB and 1,4-butanediol chain extender.

In a different approach, Chen et al. [15] have synthesized waterborne polyurethane PSAs intended for transdermal drug delivery by using the prepolymer method. Polyethylene glycol (PEG)-modified copolyether polyol and hexamethylene diisocyanate were used as reactants. They found that the holding power (related to the cohesion of the PSA), 180º peel strength and repeating peel-stick property increased as the NCO/OH ratio increased, but the tack property decreased. The optimal properties of the waterborne polyurethane PSAs, i.e., improved hydrophilicity, adhesion properties, and biocompatibility, were obtained by using NCO/OH ratios of 2.0–2.2. 

Another approach used in the development of waterborne polyurethane PSAs consisted of the synthesis of hybrid acrylic/polyurethane PSAs. Lopez et al. [16] synthesized acrylic/polyurethane hybrids with different diols chain extenders, and free radical miniemulsion polymerization was used. The authors concluded that the use of bisphenol A chain extender led to high-shear resistance PSAs with acceptable viscoelasticities. As outlined in a later publication, the same authors found an increase of the tack of the acrylic/polyurethane PSA by increasing the amount of the chain transfer agent (CTA), and this was explained to be a result of lower gel content and greater chain mobility [17]. Degrandi-Contraires et al. [18,19] proposed a different strategy in the miniemulsion polymerization of hybrid acrylic/polyurethane PSAs which consisted of the grafting of the polyurethane prepolymer onto the acrylic backbone through a reactive monomer.

The existing literature on the synthesis of waterborne polyurethane PSAs is mainly based on the use of hydroxylated polybutadienes and polypropylene glycols, cross-linkers, or hybrid acrylic/urethane polymers. All those waterborne polyurethane PSAs showed insufficient cohesion and adhesion properties, i.e., the ones showing high tack and peel strength have poor cohesion, and the ones showing high cohesion have low tack and peel strength. In our previous study [20], thermoplastic polyurethanes (TPUs) synthesized with 4,4′-diphenylmethane diisocyanate (MDI), 1,4-butanediol chain extender and mixtures of polypropylene glycols (PPGs) of different molecular weights have shown low glass transition temperatures, high tack, and low 180° peel strength, but poor cohesion. In a later study [21], TPUs with pressure-sensitive adhesion and different hard segments content were synthesized. The TPUs with low hard segments content showed high tack and adequate de-bonding properties, whereas the ones with high hard segments content increased the cohesive strength but the tack was low.

There is a need of synthesizing new polyurethane PSAs with adequate tack, peel strength and cohesion, i.e., with improved cohesion and adhesion properties. In this study, the prepolymer method was used for synthesizing different waterborne polyurethane PSAs with isophorone diisocyanate, copolymer of ether and carbonate diol polyol and different amino-alcohols chain extenders. Apart from the novelty of the use of completely new reactants for synthesizing waterborne PSAs, in this study it has been demonstrated that the number of hydroxyl groups in the amino-alcohols and the degree of cross-linking during the chain extension step facilitate the design of PSAs with different tailored properties.

## 2. Materials and Methods 

### 2.1. Materials

**Figure 1:**
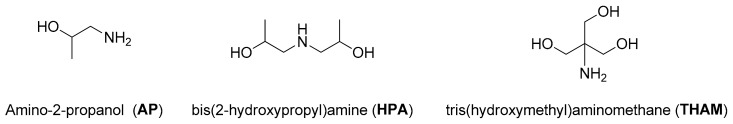
Chemical formula of the amino-alcohols used as chain extenders.

Copolymer of ether and carbonate diol (Eternacoll^®^ UT200, supplied by UBE Corporation Europe (Castellón, Spain)) with molecular weight of 2000 g/mol and functionality of 2 was used as polyol. Before synthesis, the polyol was dried in a glass flask placed in an electric blanket at 80 °C under reduced pressure (10^−3^ bars) for 2 h. Isophorone diisocyanate (IPDI, 98 wt.% purity), 2,2-bis(hydroxymethyl) propionic acid (DMPA, 98 wt.% purity) (internal emulsifier), trimethylamine (TEA, 99 wt.% purity) (neutralization agent), and dibutyltin dilaurate (DBTDL, 95 wt.% purity) catalyst were used without further purification. Three different amino-alcohols—amino-2-propanol (AP, 93 wt.% purity), bis(2-hydroxypropyl)amine (HPA, >98 wt.% purity) and tris(hydroxymethyl) aminomethane (THAM, 99.8 wt.% purity) (Figure 1)—were used as chain extenders. All those reactants were supplied by Sigma Aldrich (Sigma Aldrich Co. LLC, St. Louis, MO, USA). Methyl ethyl ketone (MEK, ACS reagent, supplied by Jaber S.A. (Almansa, Spain) and deionized water were also used.

### 2.2. Synthesis of the Waterborne Polyurethane-Urea Dispersions (WPUUs)

**Figure 2:**
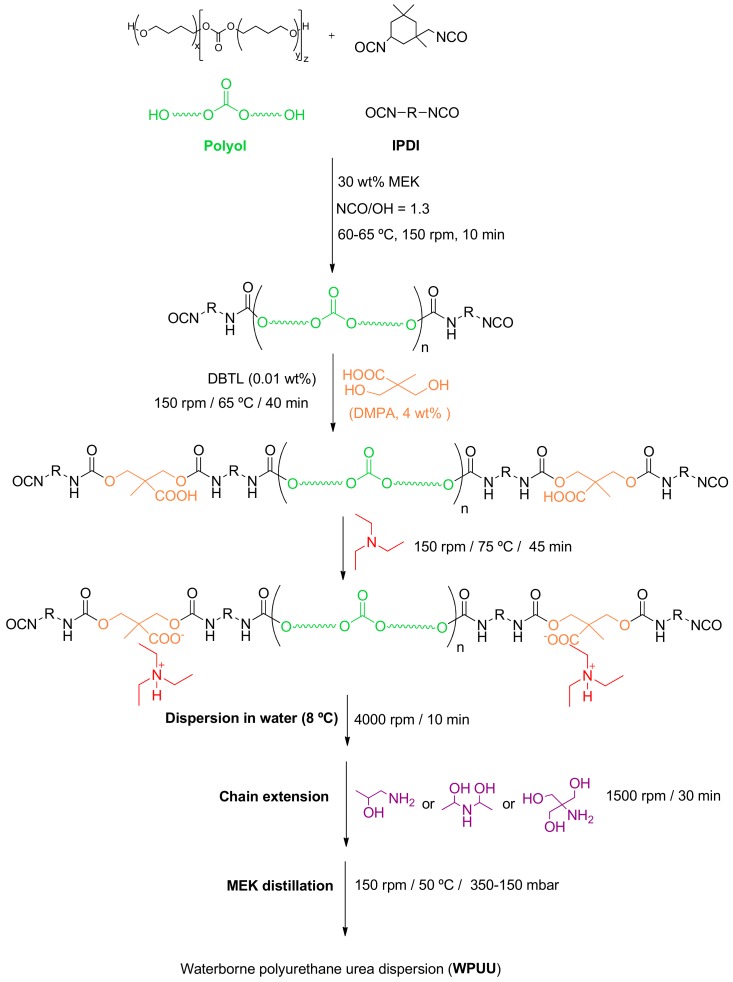
Scheme of the synthesis procedure of the Waterborne Polyurethane-Urea Dispersions (WPUUs).

The syntheses of the WPUUs were carried out in 1 L reactor by using the MEK prepolymer method (Figure 2). An NCO/OH ratio of 1.3 and 4 wt.% DMPA were used. 

The polyol and MEK (30 wt.% with respect to the prepolymer weight) were added into the reactor at 50 °C under stirring at 150 rpm. Once the mixture was homogenized, IPDI was added under stirring at 150 rpm and the temperature was increased to 60–65 °C. The stirring was maintained for 10 min. Then, DMPA was added under stirring at 150 rpm and, 10 minutes later, DBTDL catalyst (0.1 wt.% with respect to the total amount of polyol) was added. *n*-dibutylamine titration was used for determining the experimental free NCO content in the prepolymer. When the free NCO content in the prepolymer reached 7%, the required amount of TEA was added under stirring at 150 rpm and allowed to react for 45 min. Afterwards, the required amount of distilled water at 8 °C was added over the prepolymer and the stirring speed was gradually increased to 4000 rpm, and this speed was maintained for 10 min. Afterwards, the amino-alcohol chain extender was added to the prepolymer dispersed in water at 30 °C under stirring at 1500 rpm for 30 min. Finally, MEK distillation was carried out by stirring at 150 rpm at 50 °C and 100 mbar for 2 h. The nomenclature of the WPUUs were WPUU-1OH (WPUU synthesized with AP—it has 1 OH group), WPUU-2OH (WPUU synthesized with HPA—it has 2 OH groups) and WPUU-3OH (WPUU synthesized with THAM—it has 3 OH groups).

### 2.3. Preparation of the Polyurethane-Urea (PUU) Films

Solid PUU films were prepared by placing 10 g WPUU on a TQC Automatic Film Applicator (TQC Instruments, Capelle aan den Ijssel, Netherlands), a spreading rate of 20 mm/s was used. The WPUU was placed on a Teflon mould of dimensions 12 mm × 24 mm × 0.2 mm and the water was left evaporate in the open air for 24 h. Then, the PUU film was completely dried in an oven at 50 °C for 8 h. The thicknesses of the PUUs were 200–220 µm. The nomenclatures of the PUUs were PUU-1OH (PUU synthesized with AP), PUU-2OH (PUU synthesized with HPA) and PUU-3OH (PUU synthesized with THAM).

### 2.4. Preparation of the Waterborne Polyurethane Pressure-Sensitive Adhesives (PUU PSAs)

For measuring the creep resistance under shear and 180° peel strength, PUU PSAs were prepared by placing WPUU dispersion on polyethylene terephthalate (PET) film of 50 μm thick. The WPUU was applied on PET surface and it was spread with a metering rod of 200 μm, the water was evaporated at room temperature for 72 h. Polyurethane-urea films of 40–70 μm thick were obtained.

### 2.5. Experimental Techniques

#### 2.5.1. Solids Content

About 1 g waterborne polyurethane-urea dispersion was heated at 105 °C until constant weight in a DBS 60-3 thermobalance (Kern & Sohn GmbH, Balingen, Germany). Three replicates were carried out and averaged.

#### 2.5.2. pH Measurement

The pH values of the WPUUs were measured in a pHmeter HI 8418 (Oakton Instruments, Vernon Hills, IL, USA). Three replicates were carried out and averaged.

#### 2.5.3. Particle-Size Distribution 

One small droplet of WPUU was added to 1–1.5 cm^3^ distilled water and placed in Laser Scattering Particle-Size Distribution Analyser Horiba LA-950A2 (Horiba ABX SAS, Madrid, Spain) for measuring the particle-size distribution. Two replicates were measured and the mean particle sizes were averaged.

#### 2.5.4. Viscosity Measurements 

The viscosities of the WPUUs were measured at 25 °C in a DHR-2 rheometer (TA Instruments, New Castle, DE, USA), coaxial cylindrical geometry according to DIN 53019 standard was used. The cup containing the external cylinder was thermally controlled by Peltier system (Peltier Steel 999544) with 0.01 °C accuracy.

#### 2.5.5. Attenuated Total Reflection Infrared (ATR-IR) Spectroscopy

The ATR-IR spectra of the solid PUU films were obtained in a Tensor 27 FT-IR spectrometer (Bruker Optik GmbH, Erlinger, Germany), Golden Gate single reflection diamond ATR accessory was used. The angle of the incident beam was 45° and 64 scans were recorded in absorbance mode with a resolution of 4 cm^−1^.

#### 2.5.6. Differential Scanning Calorimetry (DSC)

Sealed aluminum pan containing 8.5 mg solid PUU film was placed in a DSC Q100 differential scanning calorimeter (TA Instruments, New Castle, DE, USA) and the glass transition temperatures (T_g_s) of the PUU films were measured under nitrogen atmosphere (flow rate = 100 cm^3^/min). The thermal history of the PUU films was removed by heating from −80 to 120 °C at a heating rate of 10 °C/min. After cooling down to −80 °C (cooling rate = 10 °C/min), a second DSC heating run from −80 to 250 °C was carried out by using the same heating rate. The T_g_s of the PUU films were determined from the second DSC heating runs.

#### 2.5.7. Thermal Gravimetric Analysis (TGA)

The thermal and structural properties of the PUU films were determined in a TGA Q500 equipment (TA Instruments, New Castle, DE, USA), the experiment was carried out by heating 5–10 mg PUU film up to 600 °C in nitrogen atmosphere (50 cm^3^/min); a heating rate of 10 °C/min was used.

#### 2.5.8. Plate-Plate Rheology

The viscoelastic properties of the PUU films were assessed by temperature sweep experiments in a Discovery HR-2 hybrid rheometer (TA Instruments, New Castle, DE, USA), plate–plate geometry was used. All experiments were carried out in the region of linear viscoelasticity, the gap was 0.4 mm, the frequency was 1 Hz and the strain amplitude was 2.5%. Oscillatory frequency sweep experiments at 25 °C were also carried out, and the same gap and strain amplitude were used.

#### 2.5.9. Probe Tack

For measuring the tack, WPUU was spread on stainless steel plate to obtain PUU films of 40∓70 µm thick (after water removal under open air for 48 h). The probe tack at 25 °C of the PUU films was determined in a Texture Analyzer TA.XT2i equipment (Stable Micro Systems, Surrey, UK); a cylindrical flat end steel probe of 3 mm diameter was used. For measuring the probe tack, the probe was slowly approached to the surface of the PUU film at a rate of 0.1 mm/s and, after contact, a force of 5 N was applied for 1 s, followed by pulling out at a rate of 10 mm/s. A stress-strain curve was obtained and the tack was taken at the maximum. Furthermore, the area under the stress-strain curve was calculated as it is related to the work of adhesion of the PUU film. At least three replicates for each PUU film were measured and averaged.

#### 2.5.10. Creep Resistance

**Figure 3:**
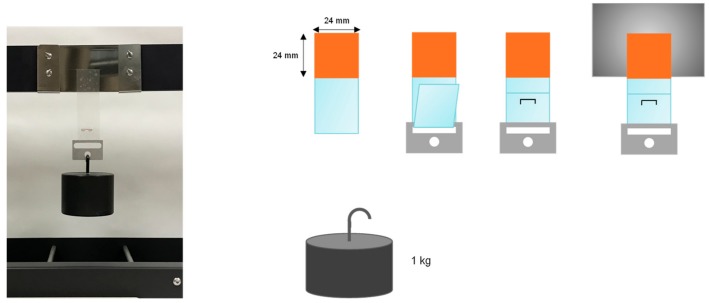
Scheme of the creep test of the polyurethane pressure sensitive adhesive (PUU PSA) and procedure for making the coupons [22].

PUU PSA, i.e., PUU on PET film, strips of 2.4 × 20 cm^2^ were placed on clean polished stainless steel 304 pieces and joined by passing a rubber coated roller of 2 kg; an area of 2.4 × 2.4 cm^2^ was joined [22]. Afterwards, the joint was placed in a Shear-10 equipment (ChemInstruments Fairfell, Fairfield, OH, USA) with a 1 kg weight grasping at the bottom. The time needed by the PUU PSA strip to fall down—“holding time”—was related to the creep resistance under shear which was measured at 25 °C and 30–50% relative humidity (Figure 3). The higher the holding time, the higher the creep resistance and the higher the cohesion of the PUU PSA. Three measurements were carried out and averaged for each PUU PSA.

#### 2.5.11. 180° Peel Test

**Figure 4:**
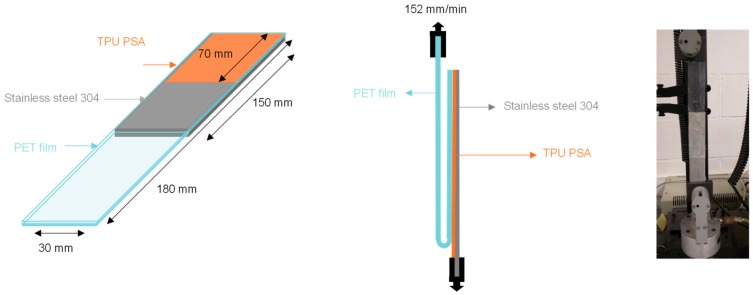
180° peel test of stainless steel 304/PUU PSA joint [22].

The adhesion of the waterborne PUU PSAs were determined by 180° peel tests of stainless steel 304/PUU PSA joints (Figure 4) in an Instron 4411 universal testing machine (Instron Ltd., Buckinghamshire, UK); a pulling rate of 152 mm/min was used. The detailed procedure for making the joints has been published elsewhere [22]. For each joint, five replicates were tested and averaged.

## 3. Results and Discussion

### 3.1. Characterization of the WPUUs

Three different WPUUs were synthetized by using three amino-alcohols with different numbers of hydroxyl groups as chain extenders (Table 1). All WPUUs synthetized with the different amino-alcohols chain extenders are translucent and stable for more than six months. The WPUUs have solids content of 38–40 wt.% (very close to the targeted solids content of 40 wt.%) and their pH values are near 9, except for WPUU-1OH dispersion which has a much higher pH (Table 1), likely due to its broader particle-size distribution—see below. 

**Figure 5:**
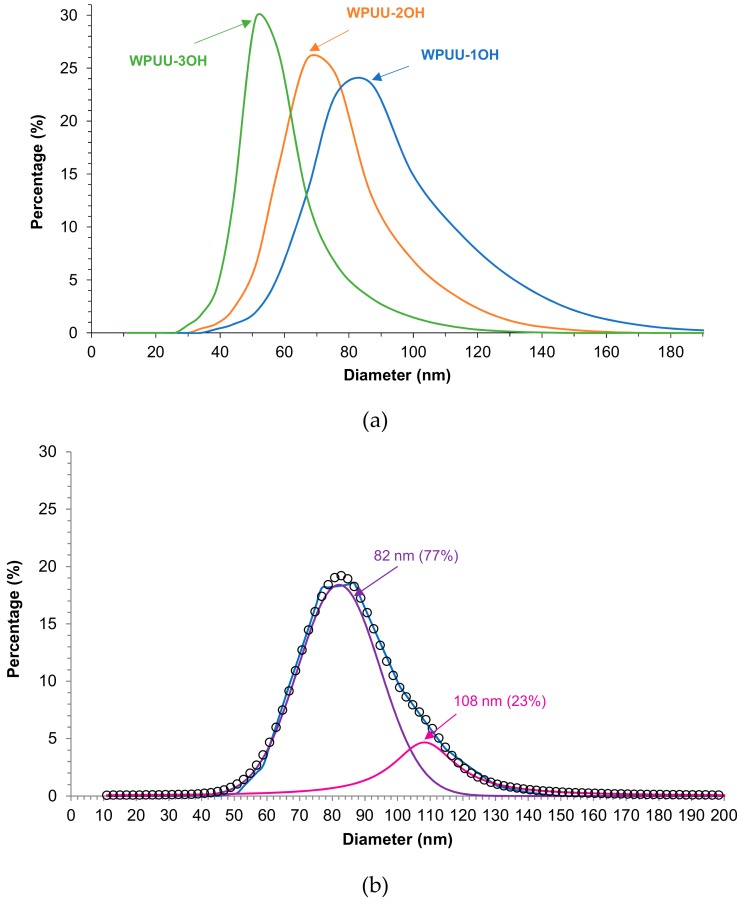
(**a**) Particle-size distributions of the WPUUs made with different amino-alcohols chain extenders. (**b**) Curve fitting of the particle-size distribution curve of WPUU-1OH.

The particle-size distributions of the WPUUs made with the different amino-alcohols chain extenders are shown in Figure 5a. All WPUUs display relatively ample tails indicating the existence of somewhat broad particle-size distributions in the range of 30–180 nm. The higher the number of the hydroxyl groups in the chain extender, the narrower the particle-size distribution of the WPUU. On the other hand, the mean particle sizes of the WPUUs range between 51 and 78 nm and they decrease by increasing the number of the hydroxyl groups in the chain extender, i.e., by increasing the degree of cross-linking during the chain extension step (Table 1). 

Because of the relatively ample tail of the particle-size distributions of the WPUUs, they were curve-fitted by using Gaussian distribution. Figure 5b shows, as typical example, the curve fitting of WPUU-1OH in which two distributions of particles with mean sizes of 82 (the most important) and 108 nm can be distinguished. The increase of the number of the hydroxyl groups of the amino-alcohol (i.e., the increase of the degree of cross-linking) decreases the particle size of both distributions in the WPUUs, and, in general, the percentage of the smaller particle sizes increases and one of the larger particles decreases by increasing the number of hydroxyl groups of the amino-alcohol (WPUU-2OH is an exception) (Table 2). Therefore, an increase of the number of the hydroxyl groups of the amino-alcohol produces narrower particle-size distributions with smaller mean particle size.

**Figure 6:**
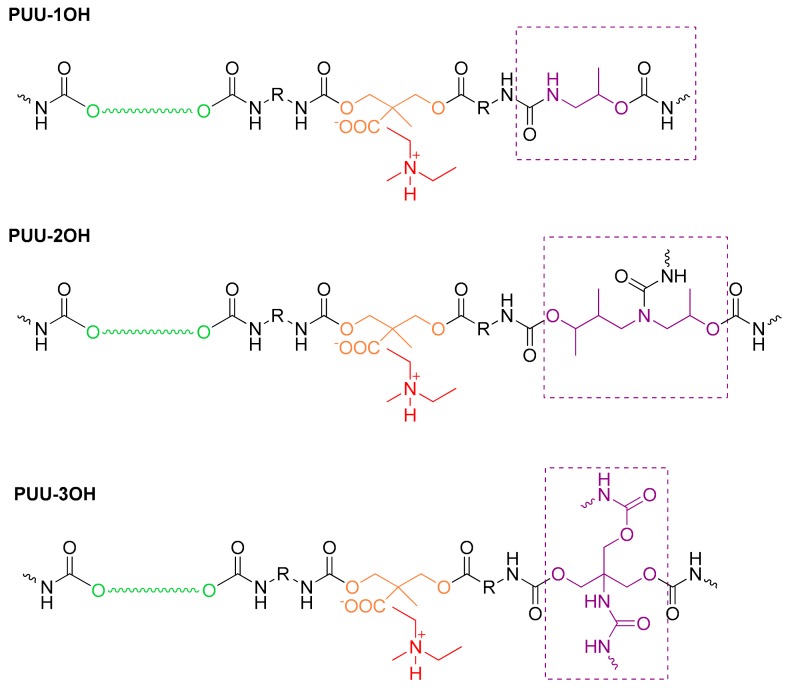
Structure of linear and cross-linked WPUUs obtained by using amino-alcohols with different number of OH groups.

It has been suggested [23] that the increasing amount of urethane groups per unit chain length reduced the polymer chains’ ability to pack closely together because the polymer chains will be expected to be more polar, leading to increased level of local interactions of physical forces, which will decrease the particle size. All WPUUs show the same structure of the soft segments consisting of IPDI-polyol-IPDI structural units, and two structural units of the hard segments corresponding to IPDI-DMPA(TEA)-IPDI, which are similar in all WPUUs, and other different structural units of IPDI-AP or IPDI-HPA or IPDI-THAM hard segments formed during the chain extension step (Figure 6). On the other hand, the structure of WPUU-1OH has the same amount of urethane and urea groups per unit chain length in the IPDI-AP hard segments, whereas the structure of WPUU-2OH has twice urethane than urea groups per unit chain length in the IPDI-HPA hard segments, and the structure of WPUU-3OH shows three-fold urethane than urea groups per unit chain length in the IPDI-THAM hard segments. Hence the smaller particle size and narrower particle-size distribution of WPUU-3OH can be associated with the increased amount of urethane groups per unit chain length produced during the chain extension step.

**Figure 7:**
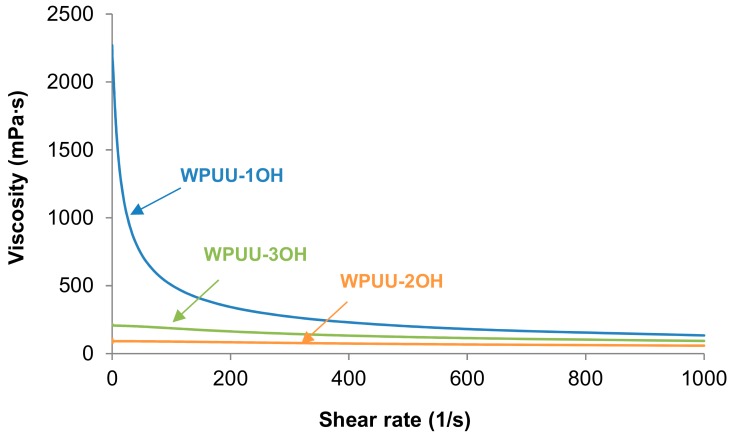
Viscosity at 25 °C of the WPUUs as a function of shear rate.

The variation of the viscosity at 25 °C as a function of the shear rate of the WPUUs (Figure 7) shows a Newtonian rheological behavior in WPUU-2OH and WPPU-3OH, and shear thinning (i.e., the viscosity decreases by increasing the shear rate) in WPUU-1OH. During shearing of the WPUU the movement of the particles of different sizes is favoured. This leads to the existence of shear thinning mainly in WPPU-1OH in which two relatively very different particle sizes coexists. Furthermore, the lack of cross-linking in WPUU-1OH may cause plastic deformation which may favour the existence of shear thinning. 

### 3.2. Characterization of the PUU Films

**Figure 8:**
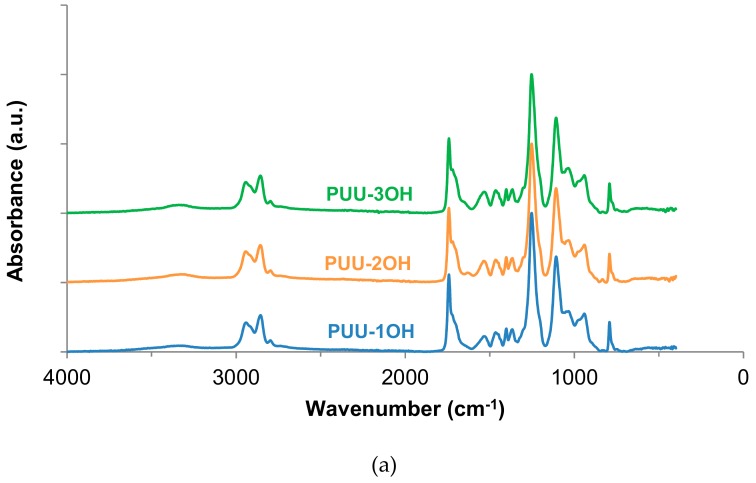

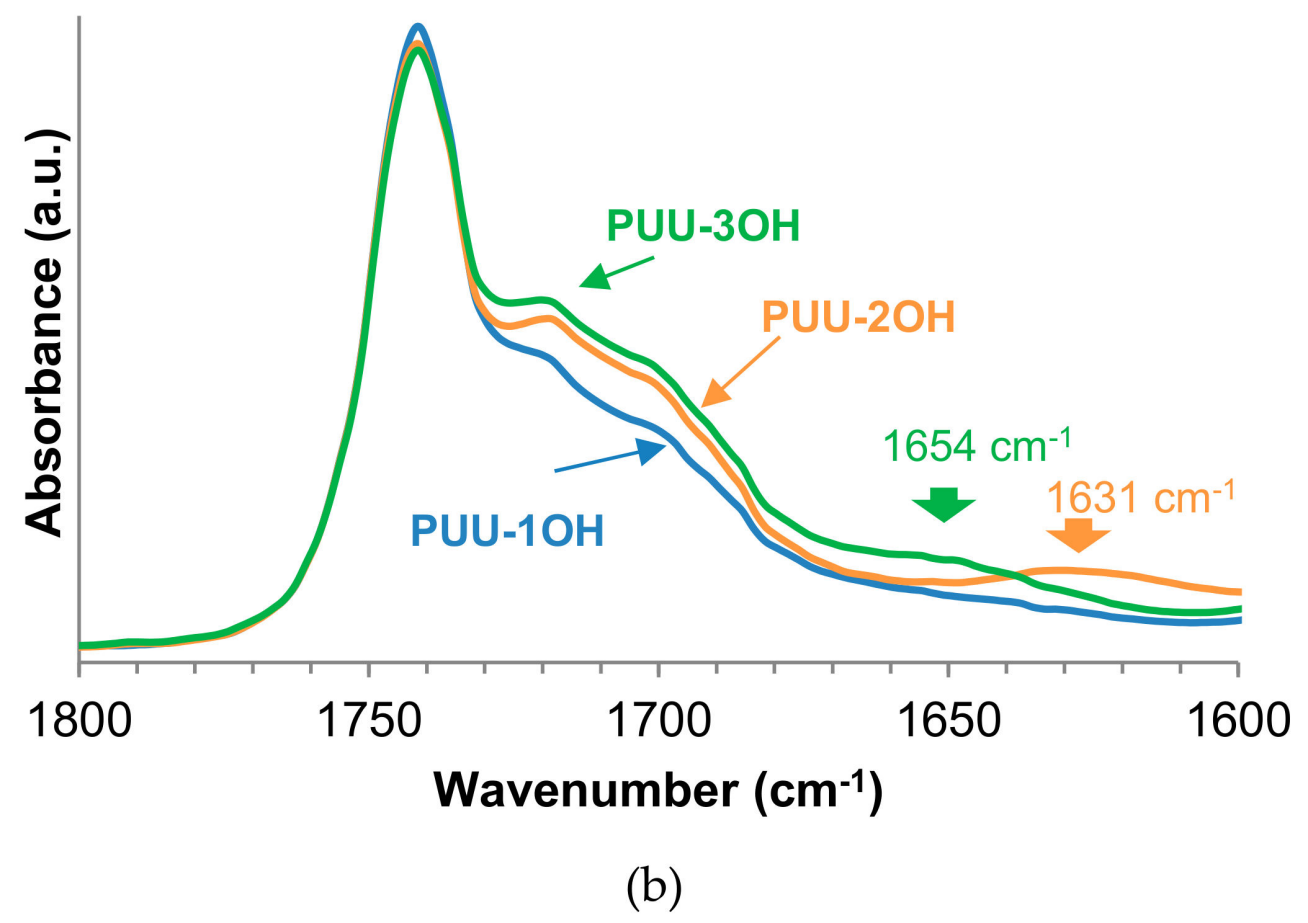
(**a**) ATR-IR spectra of the PUUs made with amino-alcohol chain extenders. (**b**) Carbonyl region (1600–1800 cm^−1^) of the ATR-IR spectra of the PUUs made with amino-alcohol chain extenders.

The chemical structure of the PUU films was assessed by ATR-IR spectroscopy. All ATR-IR spectra show similar bands (Figure 8a). The bands of the hard segments correspond to the broad N−H stretching band at 3342–3325 cm^−1^, and the N−H and C−N stretching band at 1535–1532 cm^−1^. The bands of the soft segments correspond to the aliphatic C–H stretching bands at 2943–2856 cm^−1^, and the asymmetric and symmetric −CH_2_ bending at 1465 cm^-1^ and 1402 cm^−1^ respectively. Furthermore, the C=O stretching due to the carbonate groups of the soft segments, and the urethane and urea groups of the hard segments is located at 1742 cm^−1^. On the other hand, the bands of the carbonate groups in the soft segments appear as intense bands at 1252–1253 cm^−1^ and 1036–1034 cm^−1^, and the C−O−C bands of the ether group of the polyol can be distinguished at 1108–1107 cm^−1^, 942–940 cm^-1^ and 792 cm^−1^.

**Figure 9:**
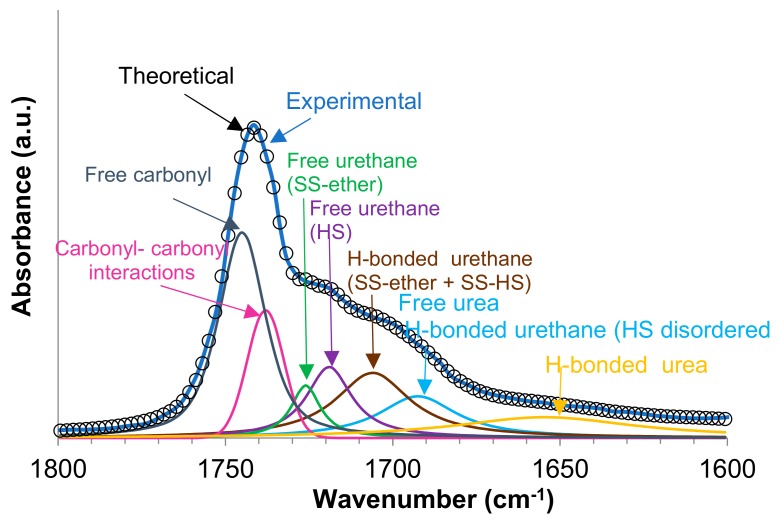
Curve fitting of the carbonyl region of PUU-1OH film.

The carbonyl region (1800-1600 cm^−1^) of the ATR-IR spectra of the PUUs shows that the bands of the urethane and urea groups are more intense by increasing the number of OH groups in the amino-alcohol (Figure 8b). Several C=O groups may contribute to the carbonyl band including the carbonate of the polyol (soft segment -SS), the carboxyl group of DMPA, and the urethane and urea hard segments (HS), as well as the interactions by hydrogen bond between them, i.e., hard segments-hard segment (HS-HS), hard segment-soft segment (HS-SS) and soft segment-soft segment (SS-SS) interactions (Figure 9) [22,24,25,26]. Because of the amounts of the DMPA and the polyol, and because the NCO/OH ratio are similar in all PUU films, the differences in the percentages of the free and associated carbonyl groups must derive from the interactions between the urethane and urea groups formed by reacting the amino-alcohol with the prepolymer during the chain extension step of the waterborne polyurethane-urea synthesis. In order to assess the compositions of the different species of C=O groups in the PUUs, the curve fitting of the carbonyl region of their ATR-IR spectra was carried out. In this study, a Gaussian distribution has been chosen and the Levenberg–Marquardt algorithm was used; the residual root mean square (RSM) fitting error was always less than 0.009.

**Figure 10:**
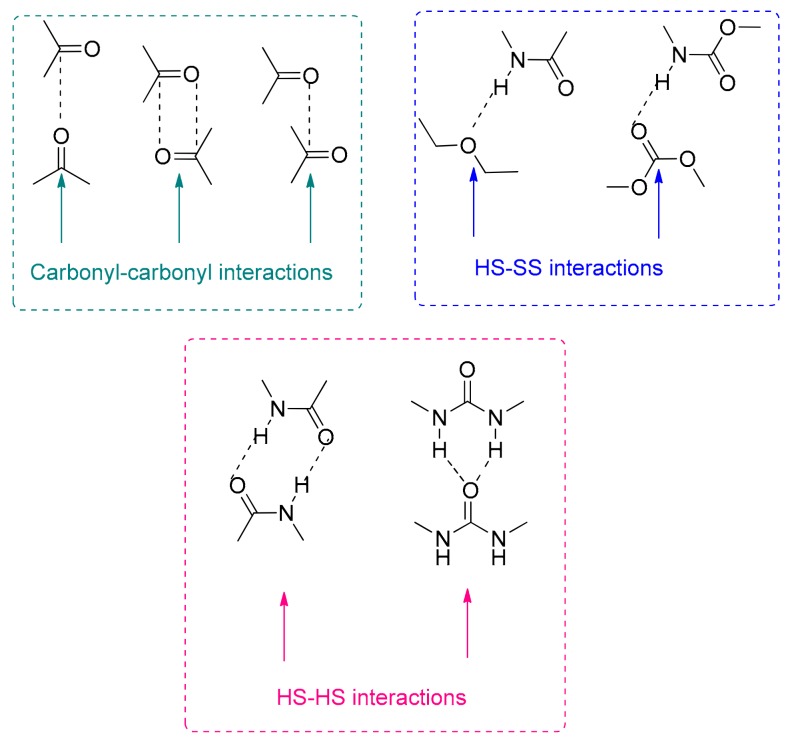
Different interactions by hydrogen bonds in the PUUs made with different chain extenders.

Figure 10 shows, as a typical example, the curve fitting of PUU-1OH film in which seven different contributions of the carbonyl groups can be distinguished [25,26]: 1745 (free carbonyl-SS), 1738 (carbonyl-carbonyl interactions-SS), 1726 (free urethane-SS ether), 1719 (free urethane-HS), 1706 (H-bonded urethane ether-SS-ether + SS-HS), 1692 (free urea + H-bonded urethane-SS-HS), and 1625–1655 (H-bonded urea-HS) cm^−1^. The percentages of each carbonyl species in the PUU films are summarized in Table 3. The more important C=O species in the PUUs are the free and associated carbonates, and their percentages decrease by increasing the number of hydroxyl groups in the amino-alcohol. On the other hand, the PUU-2OH film shows higher percentage of hydrogen bonded urea groups and lower percentages of urethane groups than the rest, indicating that its structure is different. In fact, the H-bonded urea groups appear at lower wavenumber (1625 cm^−1^) in PUU-2OH than in the other PUUs (1655 cm^−1^), indicating the existence of ordered H-bonded urea groups in PUU-2OH and the existence of disordered H-bonded urea groups in PUU-1OH and PUU-3OH [23]. The curve-fitted band at 1625–1655 cm^−1^ in the PUUs cannot be associated with unreacted NH groups because they do not appear in the ATR-IR spectra of the amino-alcohols (Figure S1 in the Supplementary Materials).

**Figure 11:**
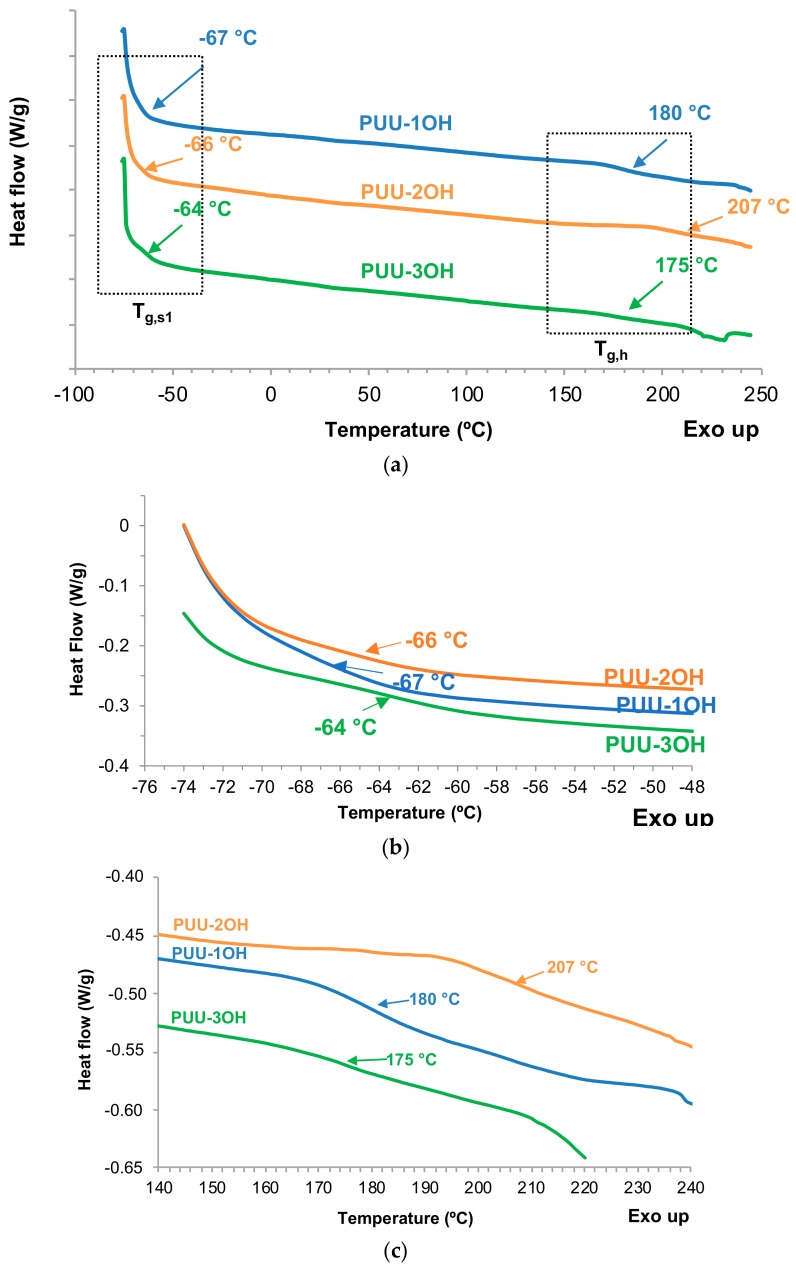
DSC thermograms of the PUU films synthesized with different amino-alcohols chain extenders. Second heating run. (**a**) Full DSC thermograms; (**b**) Region of the glass transition of the soft segments; (**c**) Region of the glass transition temperature of the hard segments.

Figure 11 shows the DSC thermograms of the PUU films. Two glass transition temperatures are observed due to the soft segments (T_g,s_) at −64 °C–−67 °C and to the hard segments (T_g,h_) at 175–207 °C. Because the polyol is the same in all PUU films, their glass transition temperatures at lower temperature are similar. The higher T_g,h_ value corresponds to the PUU-2OH film, which shows a higher degree of phase separation than the other PUU films, likely due to its higher content of hydrogen associated ordered urea hard segments. Therefore, the degree of phase separation of the PUU films depends on the number of OH functionalities of the amino-alcohol chain extender.

**Figure 12:**
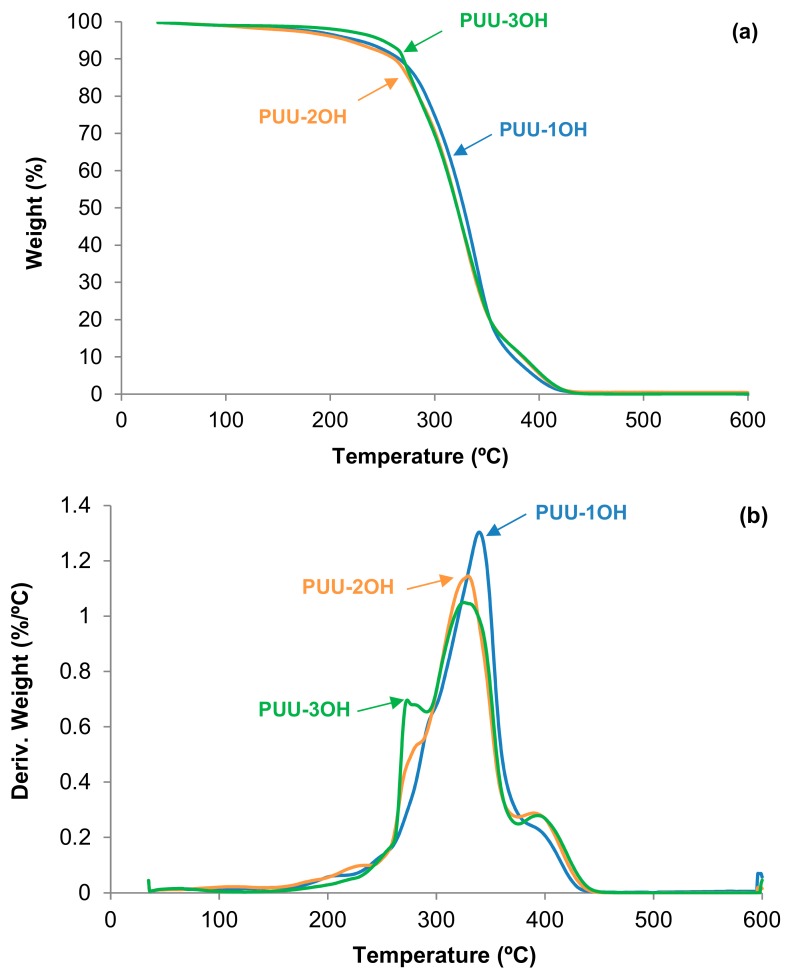
(**a**) TGA and (**b**) derivative of the TGA (DTGA) plots of PUU films made with different amino-alcohol chain extenders.

The thermal stabilities of the PUU films synthesized with different amino-alcohols chain extenders were studied by TGA. The TGA plots of the PUU films (Figure 12a) show that below 270 °C, PUU-3OH film has higher thermal stability than the rest of the PUU films and, thus, the value of the temperature at which 5 wt.% is lost (T_5%_) is higher (Table 4); however, above 270 °C, PUU-1OH film shows higher thermal stability than the other PUU films as evidenced by its higher value of the temperature at which 50 wt.% is lost (T_50%_ ). The values of T_5%_ and T_50%_ of PUU-2OH film do not follow the expected trend because its structure is different than the other PUU films. 

The derivative of the TGA thermograms of the PUU films synthesized with different amino-alcohols chain extenders (Figure 12b) shows four thermal decompositions corresponding to: (i) Urethane hard domains at 203–253 °C; (ii) Urea hard domains at 273–300 °C; (iii) Soft domains of the polyol at 325–340 °C; and (iv) soft carbonate domains at 391–395 °C (Table 5). By increasing the number of OH groups in the amino-alcohol, the decomposition of the urethane hard domains is better distinguished and they decompose at lower temperature, and both the urea hard domains and the soft domains decompose at lower temperatures. On the other hand, the amount of the soft carbonate domains slightly increases by increasing the number of OH groups in the amino-alcohol.

**Figure 13:**
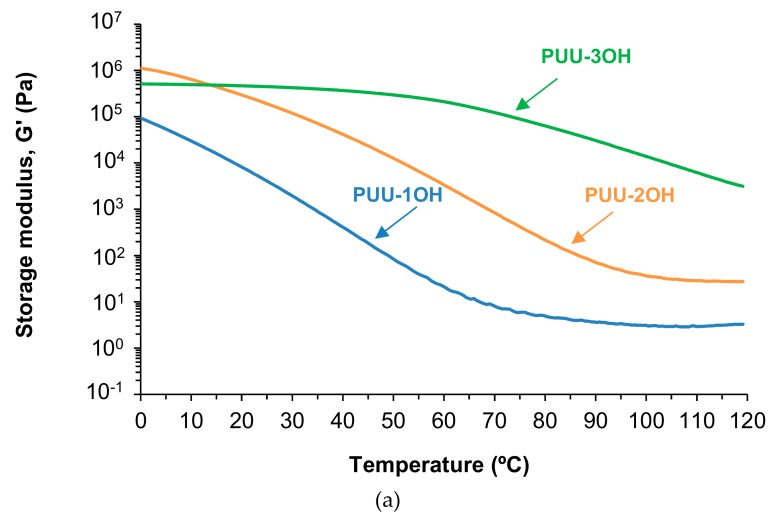

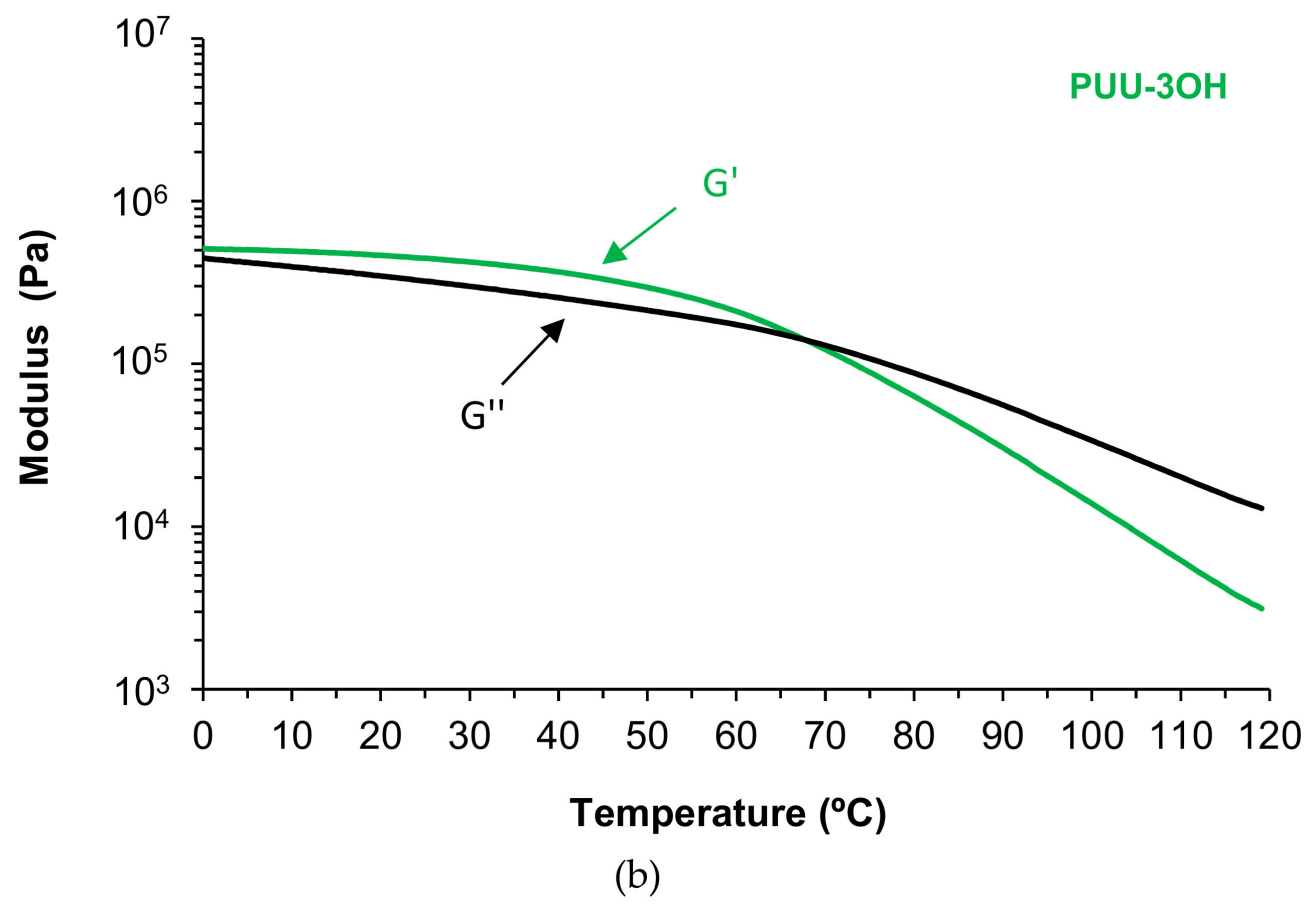
(**a**) Storage modulus (G´) as a function of the temperature for PUU films made with different amino-alcohol chain extenders. Plate-plate rheology experiments. (**b**) Variation of the storage (G´) and loss (G´´) moduli as a function of the temperature for PUU-3OH film. Temperature sweep plate-plate rheology experiments.

The viscoelastic properties of the PUU films synthesized with different amino-alcohols chain extenders were evaluated by temperature sweep plate-plate rheology experiments. Figure 13a shows that the storage moduli (G´) of the PUU films increase and the decrease of the G´ value by increasing the temperature is less marked by increasing the number of OH groups in the amino-alcohol. The increase of the degree of cross-linking during the chain extension step in the synthesis of the PUU causes an increase of the storage modulus and the mechanical properties. Figure 13b shows, as a typical example, the variation of the storage (G´) and loss (G´´) moduli as a function of the temperature for PUU-3OH film. PUU-3OH film exhibits a cross-over between G´ and G´´ moduli, i.e., above 68 °C the viscous rheological regime is dominant and below 68 °C the elastic rheological regime is dominant. At temperature below the crossing of the moduli, the values of G´ and G´´ are quite close in a broader range of temperatures, indicating an adequate balance of elastic and viscous properties. Table 6 shows that the values of the temperatures at the crossing increase and the values of the moduli at the crossing decrease by increasing the number of hydroxyl groups in the amino-alcohol chain extender; this could be related to their different degree of cross-linking during the chain extension step in the synthesis of the PUU.

### 3.3. Characterization of the PUU PSAs

PUU PSAs were prepared by placing a thin film of WPUU dispersion on PET film. The properties of the PUU PSAs are determined by their viscoelastic properties and their adhesion and cohesion properties at 25 °C.

**Figure 14:**
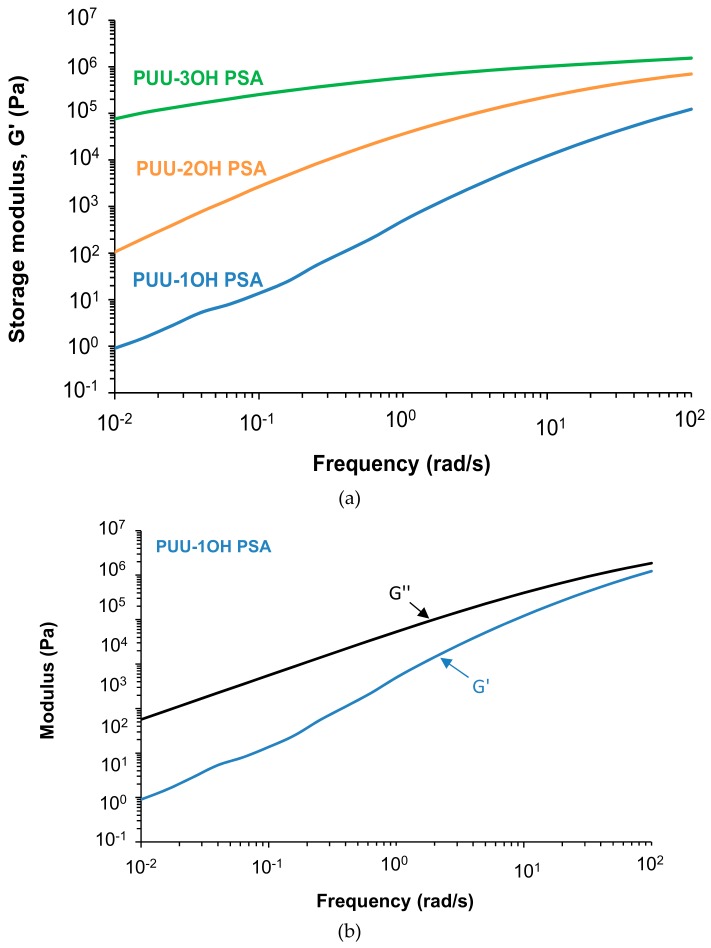

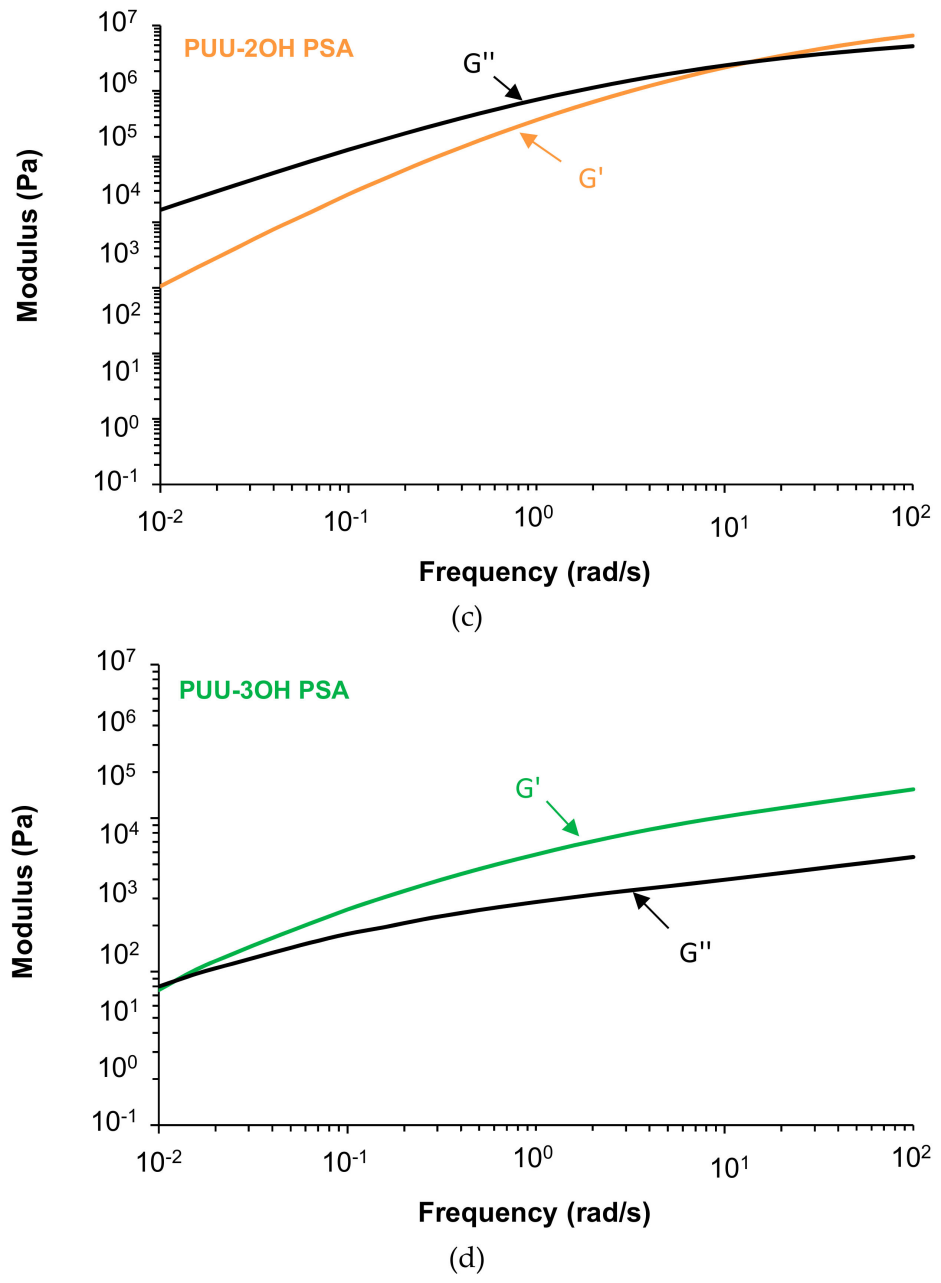
**(a)** Variation of the storage modulus (G´) as a function of the frequency for PUU PSAs made with different amino-alcohol chain extenders. Plate–plate rheology. Frequency sweep. T_ref_ = 25 °C. (b) Plots of the storage (G´) and loss (G´´) moduli as a function of the frequency for PUU PSAs made with different amino-alcohol chain extenders. Frequency sweep plate–plate rheology experiments. (**b**) PUU-1OH PSA; (**c**) PUU-2OH PSA; (**d**) PUU-3OH PSA.

Frequency sweep plate-plate rheology experiments were carried out for assessing the viscoelastic properties at 25 °C of the PUU PSAs made with different amino-alcohol chain extenders. According to Figure 14a, the variation of the storage modulus with the frequency is less important by increasing the degree of cross-linking of the PUU PSA, i.e., PUU-1OH PSA made with an amino-alcohol with one hydroxyl group shows the highest G´ value which is almost constant over all range of frequencies, whereas PUU-3OH shows the lowest G´ value at low frequencies and there is a continuous decrease of G´ with the frequency. 

According to Figure 14b, PUU-1OH PSA shows no cross-over between the storage (G´) and loss (G´´) moduli and the values of G´´ are always higher than the ones of G´, whereas in PUU-2OH PSA the cross-over appears at a frequency of 13.65 rad/s and at 0.01 rad/s in PUU-3OH PSA (Table 7). Therefore, the values of the frequency at the cross-over decreases and the G´ value increases by increasing the degree of cross-linking in the PUU PSA. The values of the storage modulus at high frequency are related to the de-bonding properties of the PSAs and the ones at low frequency are related to the bonding process of the PSAs [27]. The values of G´ at high frequency of the PUU PSAs are higher than 10^4^ Pa (Figure 14a) and, therefore, good cohesion during de-bonding can be expected in all of them. However, the values of G´ at low frequency of the PUU PSAs vary noticeably depending on the amino-alcohol chain extender and they are too low for PUU-1OH PSA and too high for PUU-3OH PSA (Figure 14a), i.e., poor cohesion can be expected during bonding of PUU-1OH PSA and poor adhesion can be expected during bonding of PUU-3OH PSA.

**Figure 15:**
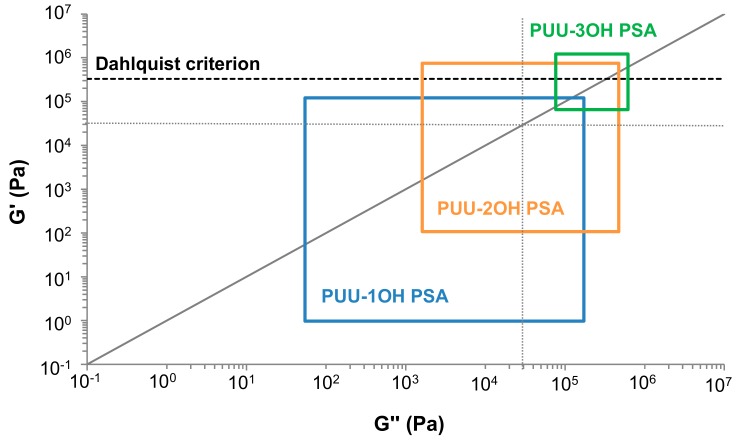
Chang’s viscoelastic windows at 25 °C of the PUU PSAs made with different amino-alcohol chain extenders. The four regions of Chang’s viscoelastic window correspond to the dotted lines.

Figure 15 shows the Chang´s viscoelastic windows of the PUU PSAs made with different amino-alcohol chain extenders; the viscoelastic windows are obtained by using the G´ and G´´ values at frequencies of 10^−2^ and 10^2^ rad/s of the viscoelastic curves of Figure 14a. The viscoelastic window of the PUU-1OH PSA corresponds to removable PSA, i.e., it has low-medium bonding modulus and low-medium dissipation [28]. The increase of the hydroxyl groups in the amino-alcohol shifts the Chang´s viscoelastic window towards the right upper corner that corresponds to high-shear PSAs, i.e., PSAs with high bonding moduli and high dissipations [28]. On the other hand, Chang´s viscoelastic window of the PUU-2OH film corresponds to general purpose PSA [28]. Therefore, different types of PSAs can be obtained by changing the number of OH groups in the amino-alcohol chain extender. Furthermore, the three PUU PSAs have the base of the viscoelastic windows located below Dahlquist’s criterion line at G´ = 3.3 × 10^5^ Pa [29]. This is indicative of a good contact of all PUU PSAs with the substrate to be bonded. 

**Figure 16:**
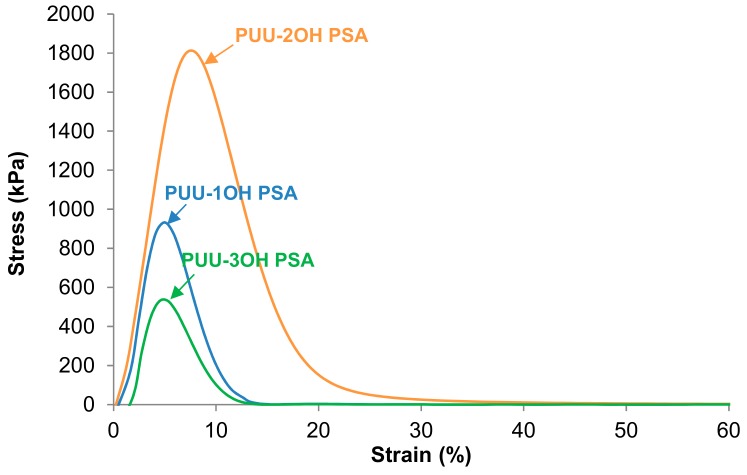
Stress-strain curves at 25 °C of the PUU PSAs made with different amino-alcohols chain extenders on stainless steel 304 plate as a function of the temperature.

The pressure-sensitive adhesives must show a good balance between their cohesion and their adhesion properties, and sufficient tack. The stress-strain curves at 25 °C of the PUU PSAs obtained during the probe tack measurement experiments are shown in Figure 16. All PUU PSAs have similar stress-strain curves and they show a relatively sharp decrease of the stress after the maximum of the curves, i.e., interfacial de-bonding of the PUU PSA from the substrate is obtained. The probe tack values at 25 °C of the PUU PSAs made with different amino-alcohol chain extenders were taken in the maximum of the stress-strain curves and they are given in Table 8. The tack at 25 °C of the PUU PSAs ranges between 488 and 1807 kPa and the highest tack values correspond to PUU-2OH PSA. PUU-2OH PSA which shows the highest degree of phase separation (Figure 11) and, therefore, the mobility of the soft segments will be higher than in the other PUU PSAs. On the other hand, the area under the stress-strain curves of Figure 16 is the work of adhesion of the PUU PSA and, according to Table 8, the highest work of adhesion corresponds to PUU-2OH PSA and the lowest one belongs to PUU-3OH PSA which shows a high G´ value at low frequency and the highest degree of cross-linking, i.e., the mobility of the soft segments is restricted, leading to low tack value too.

Table 9 shows the values of 180° peel strength of aluminum/PUU PSA joints made with the PUU PSAs. The lowest 180° peel strength value (0.4 N/cm) corresponds to the joint made with PUU-1OH PSA, due to the dominance of G´´ over G´ at 25 °C (Figure 14a), which justified the cohesive failure of the adhesive during 180° peel test. The highest 180° peel strength value (6.4 N/cm) corresponds to the joint made with PUU-2OH PSA and a cohesive failure of the adhesive is also obtained. The lowest 180° peel strength value (3.7 N/cm) corresponds to the joint made with PUU-3OH PSA and an adhesion failure to the aluminum substrate is obtained.

The cohesion or holding times at 25 °C of the PUU PSAs are given in Table 9. PUU-1OH PSA falls after 2 minutes, whereas the other PUU PSAs show higher holding time due to higher degree of cross-linking. In fact, the PUU-3OH PSA shows the highest cohesion and an excellent creep resistance as it remains adhered to the polished stainless steel plate for at least 5 days. According to Table 9, the holding time of the PUU-1OH PSA is low and part of the adhesive remains attached to the stainless steel plate, indicating poor cohesion, this low cohesion can be related to its high G´´ value (the cross-over of the G´ and G´´ moduli appears at −8 °C (Table 6)).

## 4. Conclusions

Different waterborne polyurethane-urea polymers made with amino-alcohols chain extenders with pressure-sensitive adhesive property have been synthesized. The pH values of the waterborne polyurethane-urea dispersions were near 9 except for the one made with AP chain extender. The mean particle sizes of the waterborne polyurethane-urea dispersions ranged between 51 to 78 nm, and they increased by increasing the number of OH groups in the amino-alcohol chain extender. The viscosities of the waterborne polyurethane-urea dispersions were low, and varied between 58 and 133 mPa·s. 

The more important C=O species in the PUUs were the free and associated carbonates, and their percentages decreased by increasing the number of hydroxyl groups in the amino-alcohol. The PUU-2OH film showed higher percentage of hydrogen bonded urea groups and lower percentages of urethane groups than the rest, and the H-bonded urea groups appeared at lower wavenumber, indicating that its structure was different. In fact, according to the TGA experiments, the increase of the number of OH groups in the amino-alcohol displaced the decomposition of the urethane hard domains to lower temperature, and both the urea hard domains and the soft domains decomposed at lower temperatures. On the other hand, the polyurethane-urea films showed low T_g_ value, and they had cross-over temperatures between -8 and 68 °C depending on the number of OH groups in the chain extender. 

According to Chang´s viscoelastic window, different types of PUU PSAs synthesized with different amino-alcohol chain extenders were obtained: PUU PSA synthesized with AP (one hydroxyl group) was a removable PSA, PUU PSA synthesized with HPA (two hydroxyl groups) was a general purpose PSA, and PUU PSA synthesized with THAM (three hydroxyl groups) was a high-shear PSA. All PUU PSAs showed adequate tack at 25 °C and the maximum tack was obtained in PUU-2OH PSA, the holding times of the PUU PSAs varied between 2 minutes and 5 days, and the 180° peel strength values ranged between 0.41 and 6.43 N/cm. The PUU PSAs synthesized with different amino-alcohol chain extenders have a potential for the development of new versatile waterborne polyurethane PSAs, the adhesion and cohesion properties of which can be tailored by selecting the number of OH groups in the amino-alcohol chain extender.

## Figures and Tables

**Table 1:** Nomenclature and relevant properties of the WPUUs made with amino-alcohol chain extenders.

WPUU	Solids Content ^a^ (wt.%)	pH	Mean Particle Size (nm)	Viscosity ^b^ (mPa·s)
WPUU-1OH	39.9 ± 0.8	11.3 ± 0.0	78	133
WPUU-2OH	39.7 ± 0.5	9.0 ± 0.1	66	58
WPUU-3OH	37.5 ± 0.5	9.2 ± 0.1	51	92

^a^ Theoretical targeted solids content was 40 wt.%. ^b^ Measured at 25 °C and shear rate of 1000 s^−1^.

**Table 2:** Particle sizes and percentages of particles in the WPUUs made with amino-alcohol chain extenders. Curve fittings of the particle-size distributions.

WPUU	Particle size 1 (nm)	Percentage 1 (%)	Particle size 2 (nm)	Percentage 2 (%)
WPUU-1OH	82	77	108	23
WPUU-2OH	69	67	85	33
WPUU-3OH	55	87	73	13

**Table 3:** Contributions of the different carbonyl species of the PUU films made with different amino-alcohol chain extenders. Curve fitting of the carbonyl region of the ATR-IR spectra.

Wavenumber (cm^−1^)	Percentages of Species (%)		
	PUU-1OH	PUU-2OH	PUU-3OH
1745 (Free carbonyl-SS)	30	25	24
1738 (Carbonyl-carbonyl interactions-SS)	11	10	9
1726 (Free urethane –SS ether)	5	2	5
1719 (Free urethane-HS)	11	9	12
1706 H-bonded urethane (SS-ether+ SS-HS)	17	11	22
1692 Free urea-HS + H-bonded urethane (SS-HS)	12	10	10
1655 or 1625 (H-bonded urea)	14 (1655)	32 (1625)	18 (1655)

**Table 4:** Temperatures at which 5 (T_5%_) and 50 (T_50%_) wt.% are lost for PUU films made with different amino-alcohol chain extenders. TGA experiments.

PUU Film	No. OH Groups	T_5%_ (°C)	T_50%_ (°C)
PUU-1OH	1	226	328
PUU-2OH	2	217	322
PUU-3OH	3	249	321

**Table 5:** Temperatures and weight losses of the thermal decompositions of the PUU films made with different amino-alcohol chain extenders. DTGA experiments.

PUU Film	T_1_ (°C)	Weight Loss_1_ (%)	T_2_ (°C)	Weight Loss_2_ (%)	T_3_ (°C)	Weight Loss_3_ (%)	T_4_ (°C)	Weight Loss_4_ (%)
PUU-1OH	203	8	300	17	340	66	395	9
PUU-2OH	234	7	280	14	330	67	391	12
PUU-3OH	253	6	273	19	325	62	393	13

**Table 6:** Values of the temperature (T_cross-over_) and the modulus (G_cross-over_) at the crossing of the storage and loss moduli of the PUU films made with different amino-alcohol chain extenders. Temperature sweep plate-plate rheology experiments.

PUU Film	T_cross-over_ (°C)	G_cross-over_ (kPa)
PUU-1OH	−8	239
PUU-2OH	22	242
PUU-3OH	68	140

**Table 7:** Frequency (ω_cross-over_) and modulus (G_cross-over_) values at the crossing of the storage and loss moduli of the PUU PSAs made with different amino-alcohol chain extenders. Frequency sweep plate–plate rheology experiments.

PUU PSA	ω_cross-over_ (rad/s)	G_cross-over_ (kPa)
PUU-1OH PSA	-	-
PUU-2OH PSA	13.65	277
PUU-3OH PSA	0.01	869

**Table 8:** Values of the work of adhesion and the tack at 25 °C of the PUU PSAs made with different amino-alcohol chain extenders.

PU-PSA	Work of Adhesion (J/m^2^)	Tack at 25 °C (kPa)
PUU-1OH PSA	327	926 ± 6
PUU-2OH PSA	1154	1807 ± 13
PUU-3OH PSA	171	488 ± 3

**Table 9:** 180° peel strength values of aluminum/PUU PSA joints and holding time of the PUU PSAs made with different amino-alcohols chain extenders on PET film. Locus of failure: CA = cohesive failure of the PUU PSA; A = adhesion failure to aluminum substrate.

PUU PSA	180° Peel Strength (N/cm)	Holding Time
PUU-1OH PSA	0.4 (CA)	2 ± 0 min
PUU-2OH PSA	6.4 (CA)	572 ± 157 min
PUU-3OH PSA	3.7 (A)	> 5 days

## Supplementary Materials

The following are available online at https://www.mdpi.com/1996-1944/13/3/627/s1, Figure S1. ATR-IR spectra of the amino-alcohol chain extenders.

## References

[1] Sonnenschein M.F. (2015). Polyurethanes Science, Technology, Markets, and Trends.

[2] Dieterich D. (1981). Aqueous emulsions, dispersions and solutions of polyurethanes; synthesis and properties. Prog. Org. Coat..

[3] Kim B. (1996). Aqueous polyurethane dispersions. Colloid. Polym. Sci..

[4] Noble K.L. (1997). Waterborne polyurethanes. Prog. Org. Coat..

[5] Jaudouin O., Robin J.J., Lopez-Cuesta J.M., Perrin D., Imbert C. (2012). Ionomer-based polyurethanes: A comparative study of properties and applications. Polym. Int..

[6] Yilgör I., Yilgör E., Wilkes G.L. (2015). Critical parameters in designing segmented polyurethane and their effect on morphology and properties: a comprehensive review. Polymer.

[7] Benedek I., Benedek I., Feldstein M.M. (2009). Pressure-sensitive raw materials. Handbook of Pressure Sensitive Adhesive and Products: Applications of Pressure–Sensitive Products.

[8] Czech Z., Hinterwaldner R., Benedek I., Feldstein M.M. (2009). Pressure-sensitive adhesive based on polyurethanes. Handbook of Pressure Sensitive Adhesive and Products: Applications of Pressure–Sensitive Products.

[9] Czech Z., Kócmierowska M. (2006). Water-dispersible polyurethane systems used as pressure-sensitive adhesives. Polymery.

[10] Lagiewczyk M., Czech Z. (2011). Polyurethane pressure-sensitive adhesives as raw materials for the manufacturing of protective films. Pol. J. Chem. Tech..

[11] Czech Z., Milker R., Malec A. (2006). Crosslinking of PUR-PSA waterborne systems. Rev. Adv. Mater. Sci..

[12] Akram N., Gurney R.S., Zuber M., Ishaq M., Keddie J.L. (2013). Influence of polyol molecular weight and type on the tack and peel properties of waterborne polyurethane pressure-sensitive adhesives. Macromol. React. Eng..

[13] Akram N., Zia K.M., Saeed M., Usman M., Khan W.G. (2018). Role of isophorone diisocyanate in the optimization of adhesion tendency of polyurethane pressure sensitive adhesives. J. Appl. Polym. Sci..

[14] Akram N., Zia K.M., Saeed M., Usman M., Saleem S. (2018). Impact of macrodiols on the adhesion strength of polyurethane pressure-sensitive adhesives. J. Appl. Polym. Sci..

[15] Chen X., Liu W., Zhao Y., Jiang L., Xu H., Yang X. (2009). Preparation and characterization of PEG-modified polyurethane pressure-sensitive adhesives for transdermal drug delivery. Drug. Dev. Ind. Pharm..

[16] Lopez A., Degrandi E., Canetta E., Keddie J.L., Creton C., Asua J.M. (2011). Simultaneous free radical and addition miniemulsion polymerization: Effect of the diol on the microstructure of polyurethane-acrylic pressure-sensitive adhesives. Polymer.

[17] Lopez A., Reyes Y., Degrandi-Contraires E., Canetta E., Creton C., Keddie J.L., Asua J.M. (2013). Simultaneous free radical and addition miniemulsion polymerization: Effect of the chain transfer agent on the microstructure of polyurethane-acrylic pressure-sensitive adhesives. Macromol. Mater. Eng..

[18] Degrandi-Contraires E., Lopez A., Reyes Y., Asua J.M.,  Creton C. (2013). High-shear-strength waterborne polyurethane/acrylic soft adhesives. Macromol. Mater. Eng..

[19] Degrandi-Contraires E., Udagama R., Bourgeat-Lami E., Mckenna T.F.L., Ouzineb K., Creton C. (2011). Mechanical properties of adhesive films obtained from PU−acrylic hybrid particles. Macromolecules.

[20] Fuensanta M., Martín-Martínez J.M. (2018). Thermoplastic polyurethane coatings made with mixtures of polyethers of different molecular weights with pressure sensitive adhesion property. Prog. Org. Coat..

[21] Fuensanta M., Martín-Martínez J.M. (2019). Thermoplastic polyurethane pressure sensitive adhesives made with mixtures of polypropylene glycols of different molecular weights. Int. J. Adhes. Adhes..

[22] Fuensanta M., Vallino-Moyano M.V., Martín-Martínez J.M. (2019). Balanced viscoelastic properties of pressure sensitive adhesives made with thermoplastic polyurethanes blends. Polymers.

[23] Heath R., Rungvichaniwat A. (2002). The examination of the structure property relationships of some water-dispersed polyurethane elastomers. Prog. Rubber Plast. Recycl. Technol..

[24] Strikovsky A.G., Zharkov V.V. (1993). Infra-red spectroscopy study of equilibrium association of urethane groups in poly(ether urethane)s. Polymer.

[25] Niemczyk A., Piegat A., Olalla A.S., Fray M.E. (2017). New approach to evaluate microphase separation in segmented polyurethanes containing carbonate macrodiol. Eur. Polym. J..

[26] Mattia J., Painter P. (2007). A Comparison of hydrogen bonding and order in a polyurethane and poly(urethane-urea) and their blends with poly(ethylene glycol). Macromolecules.

[27] Chu S.G., Lee L.H. (1991). Dynamic mechanical properties of pressure-sensitive adhesives. Adhesive Bonding.

[28] Chang E.P. (1997). Viscoelastic properties of pressure-sensitive adhesives. J. Adhes..

[29] Dahlquist C.A., Patrick R.L. (1969). Pressure-sensitive adhesives. Treatise on Adhesion and Adhesives.

