# Supplementary material for: New Waterborne Polyurethane-Urea Synthesized with Ether-Carbonate Copolymer and Amino-Alcohol Chain Extenders with Tailored Pressure-Sensitive Adhesion Properties
Source: Materials (Basel). 2020 Jan 31;13(3):627. doi: 10.3390/ma13030627 (PMC7040638; doi:10.3390/ma13030627)
Supplement: Supplementary file 1 [file materials-13-00627-s001.pdf]

materials

MDPI

Article

New Waterborne Polyurethane-Urea Synthesized with Ether-Carbonate Copolymer and Amino-Alcohol Chain Extenders with Tailored Pressure-Sensitive Adhesion Properties

Mónica Fuensanta [1], Abbas Khoshnood [1], Francisco Rodríguez-Llansola [2] and José Miguel Martín-Martínez [1,*]

1 Adhesion and Adhesive Laboratory, University of Alicante, 03080 Alicante, Spain; monica.fuensanta@ua.es (M.F.); abbas.khoshnood@ua.es (A.K.)
2 UBE Corporation Europe, Polígono El Serrallo, 12100 Grao Castellón, Spain; f.rodriguez@ube.com
* Correspondence: jm.martin@ua.es; Tel.: +34-9-6590-3977; Fax: +34-9-6590-9416

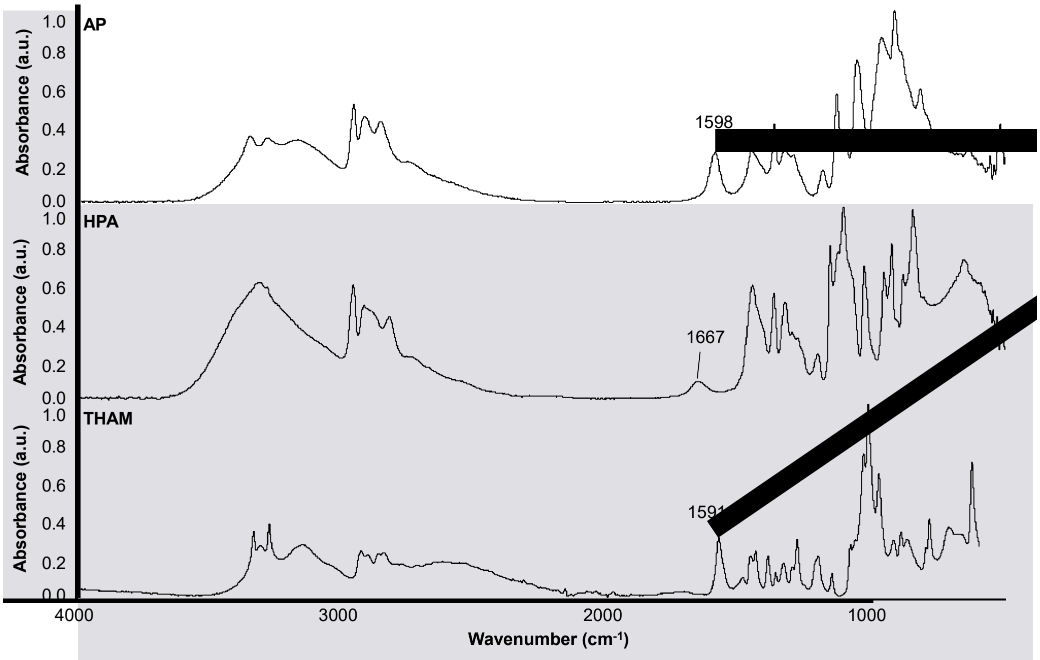

Figure 1. ATR-IR spectra of the amino-alcohol chain extenders.
